# Genetic variants of the growth differentiation factor 8 affect body conformation traits in Chinese Dabieshan cattle

**DOI:** 10.5713/ab.21.0166

**Published:** 2021-09-15

**Authors:** Shuanping Zhao, Hai Jin, Lei Xu, Yutang Jia

**Affiliations:** 1 Anhui Province Key Laboratory of Livestock and Poultry Product Safety Engineering, Institute of Animal Husbandry and Veterinary Medicine, Anhui Academy of Agricultural Sciences, Hefei 230031, China

**Keywords:** Association Analysis, Body Conformation Traits, Chinese Dabieshan Cattle, Growth Differentiation Factor 8 Gene

## Abstract

**Objective:**

The growth differentiation factor 8 (*GDF8*) gene plays a key role in bone formation, resorption, and skeletal muscle development in mammals. Here, we studied the genetic variants of *GDF8* and their contribution to body conformation traits in Chinese Dabieshan cattle.

**Methods:**

Single nucleotide polymorphisms (SNPs) were identified in the bovine *GDF8* gene by DNA sequencing. Phylogenetic analysis, motif analysis, and genetic diversity analysis were conducted using bioinformatics software. Association analysis between five SNPs, haplotype combinations, and body conformation traits was conducted in 380 individuals.

**Results:**

The GDF8 was highly conserved in seven species, and the GDF8 sequence of cattle was most similar to the sequences of sheep and goat based on the phylogenetic analysis. The motif analysis showed that there were 12 significant motifs in GDF8. Genetic diversity analysis indicated that the polymorphism information content of the five studied SNPs was within 0.25 to 0.5. Haplotype analysis revealed a total of 12 different haplotypes and those with a frequency of <0.05 were excluded. Linkage disequilibrium analysis showed a strong linkage (r^2^>0.330) between the following SNPs: g.5070C>A, g.5076T>C, and g.5148A>C. Association analysis indicated these five SNPs were associated with some of the body conformation traits (p<0.05), and the animals with haplotype combination H1H1 (-GGGG CCTTAA-) had greater wither height, hip height, heart girth, abdominal girth, and pin bone width than the other (p<0.05) Dabieshan cattle.

**Conclusion:**

Overall, our results indicate that the genetic variants of *GDF8* affected the body conformation traits of Chinese Dabieshan cattle, and the *GDF8* gene could make a strong candidate gene in Dabieshan cattle breeding programs.

## INTRODUCTION

Dabieshan cattle are a local breed, mainly raised in the central plains of China [1]. They are usually tawny, but individuals can also have black coloration. Dabieshan cattle are one of the most important local breeds in China because of the quality of their meat and adaptability. Given their longer generation times, high costs, and the increased labor they require, the growth of Dabieshan cattle by traditional breeding is slow. Body conformation traits, such as body length (BL), wither height (WH), hip height (HH), and heart girth (HG) are markedly lower in Dabieshan cattle compared with other exotic commercial cattle breeds and urgently need to be improved.

The growth and differentiation factor 8 (*GDF8*) gene, also known as myostatin, was first cloned in the mouse muscle cDNA library in 1997 [2]. Thomas et al [3] indicated that *GDF8* plays an important role in muscle differentiation and growth by inhibiting the formation and differentiation of muscle cells and impeding the growth of skeletal muscles. In Belgian Blue and Piedmontese cattle breeds, natural mutations, or deletions in *GDF8* gene have been shown to promote the proliferation and hypertrophy of muscle cells and fibers, which resulted in a 20% enhanced muscle mass [4]. In Shaanbei White Cashmere goats, a 5-bp indel in *GDF8* was shown to affect body conformation traits, such as body height, HH, and chest width index [5]. Yang [6] showed that single nucleotide polymorphisms (SNPs) in *GDF8* affected the body height, BL, HG, and hucklebone width in Nanyang, Qinchuan, and Jiaxian red cattle. These findings suggest that *GDF8* could make an excellent candidate gene for body conformation traits in livestock.

The objectives of this study were to investigate the SNPs within *GDF8*, and their associations with body conformation traits in Chinese Dabieshan cattle. The result of this study could be used to improve the design of breeding plans.

## MATERIALS AND METHODS

All procedures involving animals in this study were approved by the Animal Care and Use Committee of the Anhui Academy of Agricultural Sciences (approval number A19-CS08).

### Sequence alignment, phylogenetic analysis, and motif analysis

Amino acid sequences of *GDF8* in seven species, including cattle (*Bos taurus* NP_001001525.1), human (*Homo sapiens* NP_005250.1), rat (*Rattus norvegicus* NP_062024.1), goat (*Capra hircus* NP_001272666.1), mouse (*Mus musculus* NP_034964.1), sheep (*Ovis aries* NP_001009428.1), and pig (*Sus scrofa* NP_999600.2), were downloaded from the NCBI database. Multiple sequencing alignment was performed, and a phylogenetic tree was built for *GDF8* sequences using the neighbor-joining method in MEGA (version 7.0.26) [7]. Protein structure-function, motifs, and conserved domains were analyzed using MEME Suite [8] and NCBI Conserved Domain Database [9].

### Animals, genotypes, and phenotypes

The body conformation traits of 380 Chinese Dabieshan cattle managed in the Species Resources Protection Farm in Anhui, China, were studied. The animals were not pregnant, and all individuals were separated by more than three generations. Cattle were fed a diet based on the Nutrient Requirements of beef cattle (8th, NRC, 2016), which included 25% concentrate and 75% roughage (corn silage and dry straw) on a total mixed ration basis, along with abundant water.

Ear marginal tissues were collected from these animals. Genomic DNA was extracted using the TIANamp Genomic DNA Kit (TIANGEN, Beijing, China) and measured by a spectrophotometer. Genomic DNA was then diluted to 50 ng/μL for polymerase chain reaction (PCR) analysis. We also collected phenotypic data following the methods of Wang et al [10] and Yang et al [11]. Traits measured in this study were BL, WH, HH, HG, abdominal girth (AGR), hip width (HW), and pin bone width (PBW).

### Primers, polymerase chain reaction, and gel electrophoresis

Based on the sequence of bovine *GDF8* (GenBank accession number: NC_037329.1), two pairs of primers were designed for SNP scanning (Table 1). Five SNP sites were identified by PCR and sequencing, including two mutations in exon 1 (g.244C>G and g.400G>A), two mutations in exon 3 (g.5070C> A and g.5076T>C), and one mutation in the 3′untranslated region (UTR) (g.5148A>C). The PCR reactions were performed in a volume of 25 μL containing 20 ng of DNA template, 12.5 μL of 2×Taq Mix (including 0.1 U Taq polymerase/μL, 500 μM dNTP each, 20 mM Tris-HCl (pH8.3), 100 mM KCl, 3 mM MgCl_2_, PCR reaction enhancer, optimizer, and stabilizer, TIANGEN, China), 1.0 μL of each primer (100 ng/μL), and sterile water. DNA fragments were amplified using the following parameters: initial denaturation at 95°C for 5 minutes, 32 cycles of 94°C for 30 seconds, 56°C for 30 seconds, and 72°C for 30 seconds, followed by a final extension at 72°C for 2 minutes. These PCR products were electrophoresed on a 1.5% agarose gel stained with GeneGreen Nucleic Acid Dye (TIANGEN, China) in 1% Tris-borate-ethylenediaminetetraacetic acid buffer.

### Statistical analysis

At the five SNP sites, the genotypic and allelic frequencies were counted from the genotype data. The Hardy-Weinberg equilibrium (HWE) of alleles was examined via a χ^2^ test, which was performed using the POPGENE 3.2 software package. Population genetic indexes including gene homozygosity (H_o_), gene heterozygosity (H_e_), effective allele numbers (N_e_), and polymorphism information content (PIC) were calculated according to Chakraborty and Nei [12]. The H_o_ was calculated as −equation-, whereas H_e_ as H_e_ = 1–H_o_. Linkage disequilibrium (LD) and haplotype construction were analyzed using SHEsis software (http://analysis.bio-x.cn/myAnalysis.php) [13,14]. D’ represents the different degrees of linkage, and r^2^>0.33 indicates a strong linkage between SNP sites [15].

The mean of body conformation traits was calculated using the Bonferroni method in the SPSS 24.0 (IBM Company, NY, USA) software package. The association between SNP markers, haplotype combinations, and body conformation traits was analyzed using a general linear model with Bonferroni method as follows:


$${\text{y}}_{\text{ijk}}=\text{u}+{\text{g}}_{\text{i}}+{\text{a}}_{\text{j}}+{\text{s}}_{\text{k}}+{\text{e}}_{\text{ijk}}$$
[16]

Where y_ijk_ is the phenotypic observation, u is the population mean, g_i_ is the fixed effect of animal genotype, a_j_ is the fixed effect of age, s_k_ is the random effect of sire, and e_ijk_ is the residual error. Finally, a Bonferroni correction was performed to determine the p-value. The data were expressed as the mean±standard error, and differences were considered significant at p<0.05.

## RESULTS

### Species homology, phylogenetic tree, and motif analysis

**Figure 1:**
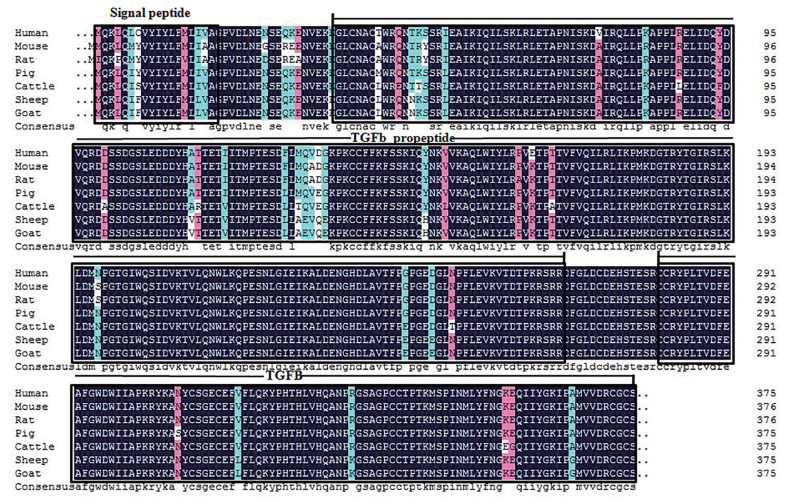
The multiple sequence alignment of growth differentiation factor 8 (GDF8) protein sequences among seven species of animals (cattle, *Bos taurus* NP_001001525.1; human, *Homo sapiens* NP_005250.1; rat, *Rattus norvegicus* NP_062024.1; goat, *Capra hircus* NP_001272666.1; mouse, *Mus musculus* NP_034964.1; sheep, *Ovis aries* NP_001009428.1; pig, *Sus scrofa* NP_999600.2). Signal peptide, starts at position 1 and ends at position 18; Pfam:TGFb_propeptide, a latent complex, consisting of the TGF-beta dimer non-covalently bound to LAP (latency associated peptide) plus a latent TGF-beta binding protein (LTBP), starts at position 37 and ends at position 266; TGFB, transforming growth factor-beta (TGF-beta) family, starts at position 281 and ends at position 375.

**Figure 2:**
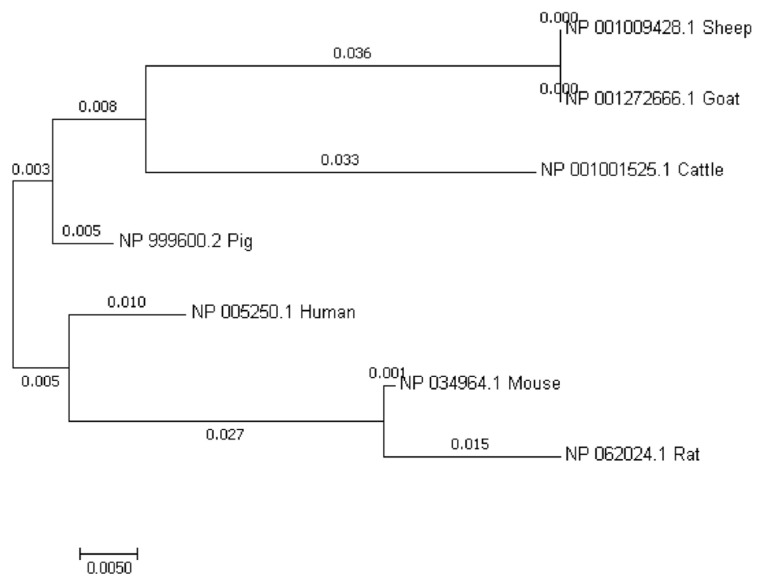
The phylogenetic tree of growth differentiation factor 8 (GDF8) protein sequences among seven species of animals (cattle, *Bos taurus* NP_001001525.1; human, *Homo sapiens* NP_005250.1; rat, *Rattus norvegicus* NP_062024.1; goat, *Capra hircus* NP_001272666.1; mouse, *Mus musculus* NP_034964.1; Sheep, *Ovis aries* NP_001009428.1; pig, *Sus scrofa* NP_999600.2).

**Figure 3:**
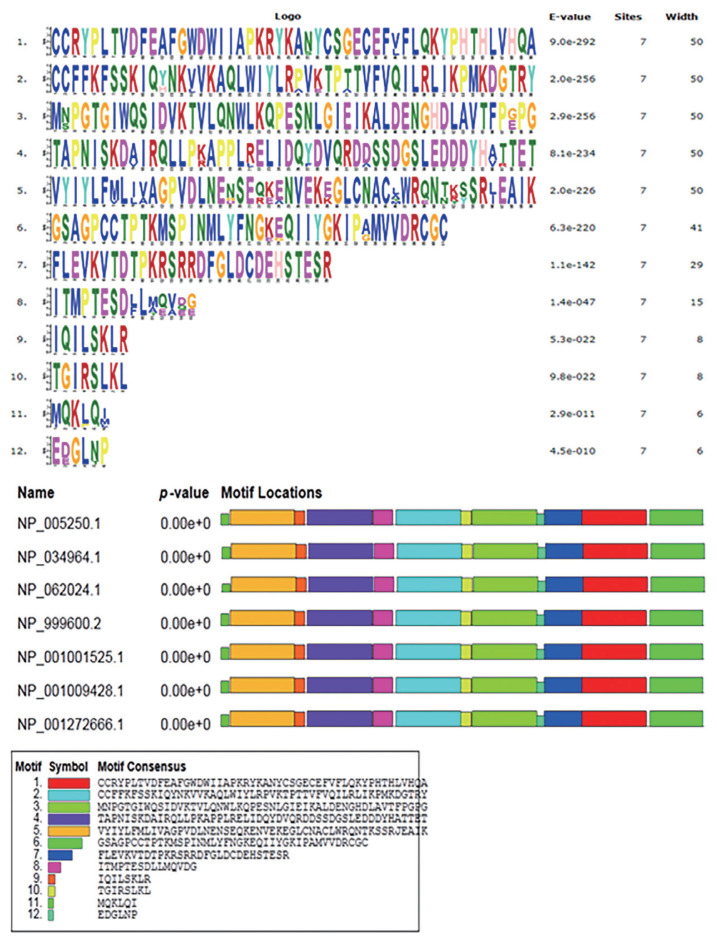
The significant motifs of growth differentiation factor 8 (GDF8) protein sequences in seven species (cattle, *Bos taurus* NP_001001525.1; human, *Homo sapiens* NP_005250.1; rat, *Rattus norvegicus* NP_062024.1; goat, *Capra hircus* NP_001272666.1; mouse, *Mus musculus* NP_034964.1; sheep, *Ovis aries* NP_001009428.1; pig, *Sus scrofa* NP_999600.2).

Our sequence analysis revealed that the cDNA in *GDF8* consisted of an 1,128-bp open reading frame, flanked by 133-bp 5′UTR and 1,476-bp 3′UTR sequences. The coding sequence regions encoded a polypeptide of 375 amino acid residues with a molecular mass of 25.74 kD and an isoelectric point of 9.58. Table 2 shows that each of the 5′-donor and 3′-acceptor splice sites conformed to the GT-AG rule. Figure 1 shows the multiple sequence alignment in seven species (cattle, human, rat, goat, mouse, sheep, and pig). Similar structures including precursor protein containing signal peptide, TGFb_propeptide, and transforming growth factor-beta (TGFB) were often observed among species. On the other hand, the phylogenic analysis of seven species revealed that cattle were closest to goat and sheep for GDF8 sequence, whereas the pig, human, rat, and mouse were relatively distant from the cattle branch. (Figure 2). A total of 12 significant motifs were identified in the related groups based on analysis of the super-secondary structure of GDF8 (Figure 3).

### Sequence variants and genetic diversity

**Figure 4:**
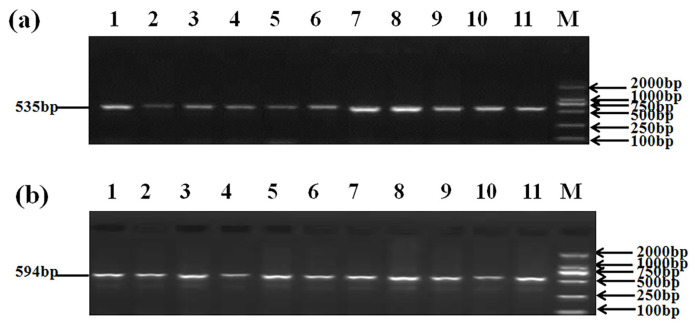
Agarose gel electrophoresis (1.5%) of polymerase chain reaction amplified product of *GDF8* gene in Dabieshan cattle specimens using (a) F1R1 primer and (b) F2R2 primer. Lanes 1 to 11 show amplification products of *GDF8* gene and Lane M shows 2,000 bp DNA ladder. *GDF8*, growth differentiation factor 8.

**Figure 5:**
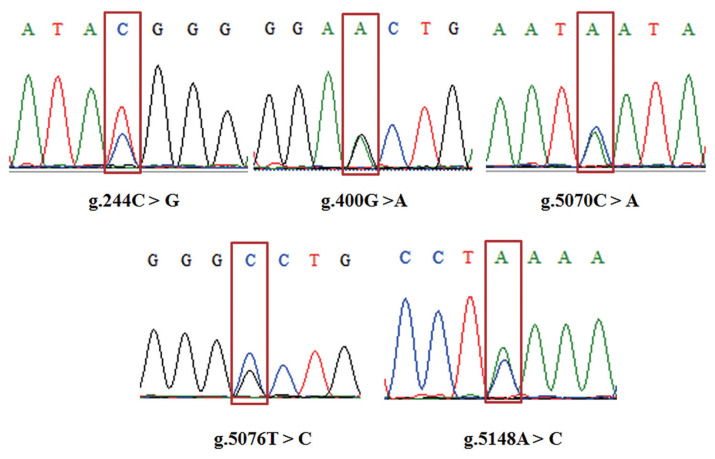
The sequenced map of five single nucleotide polymorphisms in the bovine growth differentiation factor 8 (*GDF8*) gene. The red blocks indicate the mutation site.

Five SNPs were detected in *GDF8*. Figure 4 shows the agarose gel electrophoresis (1.5%) of PCR amplified products using F1R1 and F2R2 primers; Figure 5 shows the sequenced map of five SNPs in bovine *GDF8*. In exon 1, g.244C>G and g.400G>A were detected; in exon 3, g.5070C>A and g.5076T>C were detected; in the 3′UTR, g.5148A>C was detected. Except for the SNP in the 3′UTR, the SNPs in exon1 and exon3 were synonymous mutations (g.244C>G: glycine; g.400G>A: glutamicacid; g.5070C>A: isoleucine; g.5076T>C: tyrosine).

Table 3 shows the genotype frequency, allele frequency, HWE, and population diversity parameters of five SNPs in *GDF8*. Three genotypes were observed in all five SNPs. The diversity parameters indicated that the H_e_ values ranged from 0.425 to 0.472; the range of N_e_ values was from 1.738 to 1.892, and the PIC values ranged from 0.335 to 0.360. These data indicated that Dabieshan cattle have a medium level of genetic diversity at these five SNP sites. According to the χ^2^ statistic, the genotypic frequencies of the g.244C>G, g.400G>A, and g.5148A>C mutations were in Hardy–Weinberg equilibrium. The χ^2^ statistic for g.5070C>A and g.5076T>C indicated that they were in Hardy-Weinberg disequilibrium, which may be a consequence of artificial selection and the diversity of breeding methods employed.

### Haplotype analysis

Table 4 presents the results of the LD analysis between SNP markers. We categorized r^2^ value of 0.33 or higher as strong LD. The r^2^ values between g.5070C>A, g.5076T>C and g.5148A>C in *GDF8* were greater than 0.330, indicating that the three SNPs had strong LD in Dabieshan cattle. The mean of the r^2^ values between adjacent SNPs was 0.329, and the mean of D′ values between adjacent SNPs was 0.772.

Single-site association analysis has shown that haplotypes can contribute greatly to phenotypic variation [17]. Table 5 shows the result of haplotype analysis in Dabieshan cattle. Hap 1 (-CACTA-) showed the highest frequency (32%), whereas Hap 5 (-GGACC-) showed the lowest frequency (5%) in our analysis.

### Association of markers with body conformation traits

Table 6 lists the association of SNPs with body conformation traits in Dabieshan cattle. For g.244C>G, animals with genotype GG had a significantly higher mean AGR compared with the other genotypes (p<0.05). For g.400G>A, the influence of GG genotype resulted in the highest mean HW compared with animals with genotype AA (p<0.05). For g.5070C>A site, animals carrying the AA genotype had significantly higher HH and AGR than those with the CC genotype (p<0.05). At the g.5076T>C locus, our study also found that animals with genotypes TT and CC had greater WH than animals with genotype TC (p<0.05), and animals with the genotype CC had greater HH than animals with other genotypes (p<0.05). At the g.5148A>C locus, animals with genotype CC exhibited greater HH than the animals with genotype AA and AC (p<0.05).

### Association of combined haplotypes with body conformation traits

Table 7 shows the association of combined haplotypes with body conformation traits in Dabieshan cattle. The frequencies of combined haplotypes <5.0% were not considered. Dabieshan cattle with H1H1 resulted in the highest means for WH, HH, HG, AGR, and PBW compared with animals with other combined haplotypes (p<0.05).

## DISCUSSION

In China, Dabieshan cattle are generally considered as an economically important local breed that is extensively farmed and produces a high quality product [1]. *GDF8* has been shown to possess complex biological functions, especially in muscular development [3,4], bone metabolism [18], and body development. Many studies have shown that SNPs in *GDF8* are associated with body conformation traits in goat [19], yak [20], horse [21], and cattle [6].

In this study, multiple sequence alignment indicated that *GDF8* was highly homologous in seven species, suggesting that it may possess critically important functions. The results of the phylogenic analysis in this study were consistent with Wu et al [22], which showed that the GDF8 sequence of cattle was most like sheep and goat. Three genotypes were identified for the five SNPs, and the PIC indicated that *GDF8* showed an intermediate level of polymorphism (0.25<PIC<0.5) in Chinese Dabieshan cattle. The r^2^-values above 1/3 (r^2^>0.33) indicate that LD was sufficiently strong for mapping [15]. The mean r^2^ indicated that LD among the g.5070C>A, g.5076T>C, and g.5148A>C sites was strong, and this may be attributed to the lower recombination and higher genotypic variation at these sites [23].

It was worth noting that, mutations of g.244C>G, g.400G>A, g.5070C>A, and g.5076T>C were synonymous polymorphisms in Dabieshan cattle. Our results were consistent with the study of Huang et al [24], which found that a silent mutation of the bovine sterol regulatory element-binding protein-1c (*SREBP1c*) gene was associated with cattle body weight. Our results were also consistent with those of Xu et al [25] who found that a ‘silent’ SNP (g. 4617 A>C) in PAX3 dramatically improves the WH and BL of Chinese Nanyang and Caoyuan cattle. As Hunt et al [26] reported, a silent mutation did not alter the protein primary structure; however, this mutation might affect protein folding, alter the function, and modify the cellular response to specific targets by affecting messenger RNA splicing, stability, and protein structure. The SNPs in the 3′ UTR may be within or in the vicinity of the microRNA (miRNA) binding site, which could impair the regulatory functions of the associated miRNA [27,28]. Bioinformatics analysis in the current study showed that g.5148A>C was upstream of the bta-miR-29abcd binding site (http://www.targetscan.org/vert/); this may alter the level of *GDF8* gene expression and affect phenotypes [29,30]. These findings were consistent with studies of Guanzhong dairy goats [31].

The bovine *GDF8* gene is localized on chromosome 2 and has three exons and two introns. In this study, we detected five SNPs in bovine *GDF8* gene: g.244C>G, g.400G>A, g.5070C>A, g. 5076T>C, and g.5148A>C. Data analysis revealed that all five SNPs were significantly associated with their body conformation traits, and animals with the combined haplotype H1H1 (-GGGGCCTTAA-) had significantly improved body conformation traits than animals with other haplotypes. Hap1 (-CACTA-) might also be associated with enhanced body conformation traits in Chinese Dabieshan cattle. Hence, we suggest that the combined haplotype H1H1 (-GGGGCCTTAA-) could be used as a marker to aid the selection of desirable characteristics of Dabieshan cattle in breeding programs.

## CONCLUSION

Dabieshan cattle are economically important local breed in China. In this study, five SNPs and their corresponding haplotypes were identified in Chinese Dabieshan cattle, and the association analysis revealed that all five loci were significantly associated with their body conformation traits. Our results indicated that the combined haplotype H1H1 (-GG GGCCTTAA-) could be used as a marker to improve the body conformation traits of Dabieshan cattle in breeding program. Given the complexity of cattle breeding, additional studies are needed to examine the functional effects of *GDF8* functional effects on body conformation traits.

## Figures and Tables

**Table 1:** Primers used for polymerase chain reaction analysis in bovine GDF8

Primer name^1)^	Primer sequence	Annealing temperature (°C)	Segment length (bp)
*GDF8*-F1	5′ –GGAAGAAGTAAGAACAAGGGA– 3′	56	535
*GDF8*-R1	5′ – TCCTCCTTACATACAAGCCAG– 3′		
*GDF8*-F2	5′ –TCTTCTTTCCTTTCCATACAG– 3′	56	594
*GDF8*-R2	5′ –TACGATTTGTTTTGATGGTTA– 3′		

GDF8, growth differentiation factor 8.

1) GDF8-F*, for forward; GDF8-R*, for reverse.

**Table 2:** The exon/intron organizations of bovine *GDF8*

Gene	Number	Exon size (bp)	Intron size (bp)	5′ splice donor	3′ splice acceptor
*GDF8*	1	373	1,828	GGAGTgtgag	tatagCTGAT
	2	374	2,032	GACTGgtaag	tacagACTCC
	3	381	-	-	-

*GDF8*, growth differentiation factor 8.

**Table 3:** Population genetic diversity for SNPs in Dabieshan cattle

SNP	Genotypic frequency			Allelic frequency		HWE			Diversity parameter		
			
	1	2	3	A1	A2	χ^2^	p-value	H_o_	H_e_	N_e_	PIC
SNP1 (g.244C>G)	CC	GC	GG	C	G						
	0.12	0.42	0.46	0.33	0.67	0.29	0.867	0.560	0.440	1.786	0.343
SNP2 (g.400G>A)	GG	GA	AA	G	A						
	0.10	0.43	0.47	0.32	0.68	0.06	0.972	0.575	0.425	1.738	0.335
SNP3 (g.5070C>A)	CC	CA	AA	C	A						
	0.44	0.36	0.20	0.62	0.38	13.46	0.001	0.528	0.472	1.892	0.360
SNP4 (g.5076T>C)	TT	TC	CC	T	C						
	0.50	0.34	0.16	0.67	0.33	13.91	0.001	0.557	0.443	1.795	0.345
SNP5 (g.5148A>C)	AA	AC	CC	A	C						
	0.46	0.38	0.16	0.65	0.35	6.00	0.051	0.544	0.456	1.837	0.352

SNP, single nucleotide polymorphism; HWE, hardy-Weinberg equilibrium; χ^2^ statistic, Chi-square test value; H_o_, genetic homozygosity; H_e_, genetic heterozygosity; N_e_, effective number of alleles; PIC, polymorphic information content.

**Table 4:** The result of linkage equilibrium analysis between SNP markers

Item	D'	r^2^
SNP1–SNP2 (g.244C>G - g.400G>A)	0.943	0.206
SNP1–SNP3 (g.244C>G - g.5070C>A)	0.780	0.191
SNP1–SNP4 (g.244C>G - g.5076T>C)	0.835	0.175
SNP1–SNP5 (g.244C>G - g.5148A>C)	0.815	0.188
SNP2–SNP3 (g.400G>A - g.5070C>A)	0.372	0.038
SNP2–SNP4 (g.400G>A - g.5076T>C)	0.533	0.062
SNP2–SNP5 (g.400G>A - g.5148A>C)	0.572	0.081
SNP3–SNP4 (g.5070C>A - g.5076T>C)	0.966	0.748
SNP3–SNP5 (g.5070C>A - g.5148A>C)	0.917	0.758
SNP4–SNP5 (g.5076T>C - g.5148A>C)	0.959	0.843

SNP, single nucleotide polymorphism.

The value of D'/r^2^ represents the degree of linkage between SNPs markers.

D' = 1, absolute linkage; D' = 0, linkage equilibrium; 0<D'<1, different degrees of linkage; r^2^>0.33 indicates a strong linkage between SNP markers.

**Table 5:** Haplotypes and their frequencies in Dabieshan cattle

Haplotype	g.244C>G	g.400G>A	g.5070C>A	g.5076T>C	g.5148A>C	Frequency (%)
Hap1	C	A	C	T	A	31.50
Hap2	G	A	A	C	C	23.80
Hap3	G	G	C	T	A	23.50
Hap4	G	A	C	T	A	5.80
Hap5	G	G	A	C	C	5.20

Hap1, CACTA; Hap2, GAACC; Hap3, GGCTA; Hap4, GACTA; Hap5, GGACC.

Haplotype with frequency <5.00% was ignored in analysis.

**Table 6:** Association of SNPs with body conformation traits in Dabieshan cattle

SNP	Genotype	Traits (mean±SE)						

		BL (cm)	WH (cm)	HH (cm)	HG (cm)	AGR (cm)	HW (cm)	PBW (cm)
SNP1 (g.244C > G)	CC	128.07±1.03	110.90±0.57	109.89±0.49	147.97±0.93	163.79±1.54^a^	30.89±0.47	17.26±0.21
	GC	126.56±0.91	110.37±0.59	109.00±0.51	148.21±0.93	167.17±1.32^a^	31.18±0.43	16.69±0.15
	GG	125.39±1.05	109.40±0.66	110.80±0.58	148.39±1.72	171.78±1.70^b^	32.16±0.42	18.73±0.93
	p-value	0.385	0.376	0.127	0.987	0.015	0.244	0.200
SNP2 (g.400G > A)	GG	130.48±1.13	112.26±0.58	111.58±0.51	151.91±1.26	173.96±1.62	32.47±0.45^a^	18.37±0.28
	GA	125.95±0.99	110.07±0.59	110.21±0.53	149.65±1.05	168.99±1.60	32.31±0.43^a^	18.58±1.12
	AA	125.28±0.95	109.45±0.65	109.35±0.62	147.22±0.94	167.82±1.46	30.78±0.40^b^	16.89±0.27
	p-value	0.070	0.140	0.257	0.058	0.216	0.034	0.292
SNP3 (g.5070C > A)	CC	125.54±1.04	110.65±0.53	109.70±0.49^a^	149.47±1.06	165.80±1.52^a^	31.77±0.44	19.23±1.26
	CA	126.51±0.95	109.26±0.69	109.71±0.53^a^	149.59±0.93	170.32±1.66^ab^	31.55±0.43	17.47±2.16
	AA	125.82±0.97	109.77±0.74	112.54±0.76^b^	149.86±1.08	175.10±1.48^b^	31.10±0.45	17.40±1.15
	p-value	0.808	0.344	0.028	0.980	0.004	0.755	0.372
SNP4 (g.5076T > C)	TT	126.11±1.09	110.92±0.60^a^	110.03±0.49^a^	149.79±1.02	167.31±1.53	31.82±0.44	18.92±1.14
	TC	126.16±0.98	108.55±0.64^b^	109.68±0.53^a^	149.30±0.99	170.53±1.72	31.54±0.45	17.52±1.24
	CC	125.08±0.97	110.00±0.77^a^	112.83±0.82^b^	149.50±1.11	173.31±1.51	31.06±0.48	17.22±1.23
	p-value	0.846	0.040	0.033	0.947	0.109	0.718	0.461
SNP5 (g.5148A > C)	AA	126.21±0.99	110.96±0.52	110.23±0.47^a^	150.19±1.06	167.19±1.59	32.25±0.44	19.19±1.22
	AC	125.94±1.03	108.74±0.67	109.60±0.54^a^	148.72±0.95	169.99±1.60	31.15±0.43	17.44±1.24
	CC	125.29±0.98	110.11±0.79	112.80±0.83^b^	149.94±1.14	174.17±1.53	31.10±0.48	17.26±1.23
	p-value	0.894	0.052	0.036	0.605	0.075	0.280	0.347

SNP, single nucleotide polymorphism; BL, body length; WH, wither height; HH, hip height; HG, heart girth; AGR, abdominal girth; HW, hip width; PBW, pin bone width.

a,b Means in the same column with different superscripts are significantly different (p<0.05).

**Table 7:** Associations of combined haplotype with body conformation traits in Dabieshan cattle

Combined haplotype	Frequency	BL (cm)	WH (cm)	HH (cm)	HG (cm)	AGR (cm)	HW (cm)	PBW (cm)
GCAAACCTAC (H1H2)	0.323	125.93±0.89	108.88±1.01^b^	110.01±0.97^ab^	148.36±1.21^b^	168.14±1.35^b^	30.33±0.86	17.19±0.50^ab^
GCAGCCTTAA (H1H3)	0.231	125.79±0.88	110.12±1.09^ab^	107.85±0.74^b^	149.27±1.47^b^	161.08±1.36^b^	31.69±0.57	16.77±0.64^b^
GGAAAACCCC (H2H2)	0.138	126.44±1.19	110.44±0.97^ab^	110.00±1.03^ab^	151.69±1.29^ab^	170.53±1.37^ab^	32.77±0.94	19.00±0.57^a^
CCAACCTTAA (H3H3)	0.121	128.86±0.95	110.29±0.95^ab^	108.83±0.81^b^	148.00±0.89^b^	166.05±1.56^b^	30.92±0.77	17.33±0.66^ab^
GGGGCCTTAA (H1H1)	0.104	128.11±1.01	112.17±0.88^a^	112.73±0.94^a^	154.06±1.16^a^	174.00±1.76^a^	33.38±0.48	19.00±0.64^a^
p-value		0.510	0.036	0.043	0.030	0.029	0.703	0.042

BL, body length; WH, wither height; HH, hip height; HG, heart girth; AG, abdominal girth; HW, hip width; PBW, pin bone width.

a,b Means in the same column with different superscripts indicate significant difference at p<0.05.

## References

[1] (2010). China national commission of animal genetic resources. Animal genetic research in China, bovines.

[2] McPherron A, Lawler AM, Lee S (1997). Regulation of skeletal muscle mass in mice by a new TGF-beta superfamily member. Nature.

[3] Thomas M, Langley B, Berry C (2000). Myostatin, a negative regulator of muscle growth, functions by inhibiting myoblast proliferation. J Biol Chem.

[4] Grobet L, Martin LJ, Poncelet D (1997). A deletion in the bovine myostatin gene causes the double-muscled phenotype in cattle. Nat Genet.

[5] Bi Y, Feng B, Wang Z (2020). Myostatin (MSTN) gene indel variation and its associations with body traits in Shaanbei White Cashmere goat. Animals (Basel).

[6] Yang DY (2006). Study on molecular markers of growth and development in cattle by candidate genes [Doctor’s thesis].

[7] Saitou N, Nei M (1987). The neighbor-joining method: a new method for reconstructing phylogenetic trees. Mol Biol Evol.

[8] Bailey TL, Boden M, Buske FA (2009). MEME SUITE: tools for motif discovery and searching. Nucleic Acids Res.

[9] Marchler-Bauer A, Bo Y, Han L (2016). CDD/SPARCLE: functional classification of proteins via subfamily domain architectures. Nucleic Acids Res.

[10] Wang G, Zhang S, Wei S (2014). Novel polymorphisms of SIX4 gene and their association with body measurement traits in Qinchuan cattle. Gene.

[11] Yang W, Wang Y, Fu C, Zan LS (2015). Association study and expression analysis of MTNR1A as a candidate gene for body measurement and meat quality traits in Qinchuan cattle. Gene.

[12] Chakraborty R, Nei M (1977). Bottleneck effects on average heterozygosity and genetic distance with the stepwise mutation model. Evolution.

[13] Slatkin M (2008). Linkage disequilibrium—understanding the evolutionary past and mapping the medical future. Nat Rev Genet.

[14] Li Z, Zhang Z, He Z (2009). A partition-ligation-combination-subdivision EM algorithm for haplotype inference with multiallelic markers: update of the SHEsis (http://analysis.bio-x.cn). Cell Res.

[15] Ardlie KG, Kruglyak L, Seielstad M (2002). Patterns of linkage disequilibrium in the human genome. Nat Rev Genet.

[16] Gui LS, Raza SHA, Jia J (2019). Analysis of the oxidized low density lipoprotein receptor 1 gene as a potential marker for carcass quality traits in Qinchuan cattle. Asian-Australas J Anim Sci.

[17] Akey J, Jin L, Xiong M (2001). Haplotypes vs single marker linkage disequilibrium tests: what do we gain?. Eur J Hum Genet.

[18] Chen YS, Guo Q, Guo LJ (2017). GDF8 inhibits bone formation and promotes bone resorption in mice. Clin Exp Pharmacol Physiol.

[19] Na R, Ni WW, EGX, Zeng Y, Han YG, Huang YF (2020). SNP screening of the MSTN gene and correlation analysis between genetic polymorphisms and growth traits in Dazu black goat. Anim Biotechnol.

[20] Liang CN, Yan P, Xing CF (2011). Association of single nucleotide polymorphism at intron 2 of MSTN gene with growth traits in yak. Journal of Huazhong Agricultural University.

[21] Tozaki T, Sato F, Hill EW (2011). Sequence variants at the myostatin gene locus influence the body composition of Thoroughbred horses. J Vet Med Sci.

[22] Wu S, Ning Y, Raza SHA (2019). Genetic variants and haplotype combination in the bovine CRTC3 affected conformation traits in two Chinese native cattle breeds (Bos Taurus). Genomics.

[23] Wen YF, Zheng L, Niu H (2020). Exploring genotype-phenotype relationships of the CRABP2 gene on growth traits in beef cattle. Anim Biotechnol.

[24] Huang YZ, He H, Sun JJ (2011). Haplotype combination of SREBP-1c gene sequence variants is associated with growth traits in cattle. Genome.

[25] Xu Y, Cai H, Zhou Y (2014). SNP and haplotype analysis of paired box 3 (PAX3) gene provide evidence for association with growth traits in Chinese cattle. Mol Biol Rep.

[26] Komar A (2009). Single nucleotide polymorphisms. Methods in molecular biology (Methods and Protocols).

[27] Nakano M, Mohri T, Fukami T (2015). Single-nucleotide polymorphisms in cytochrome P450 2E1 (CYP2E1) 3′-untranslated region affect the regulation of CYP2E1 by miR-570. Drug Metab Dispos.

[28] Yie SM, Li LH, Xiao R, Librach CL (2008). A single base-pair mutation in the 3′-untranslated region of HLA-G mRNA is associated with preeclampsia. Mol Hum Reprod.

[29] Schwerin M, Maak S, Hagendorf A, Lengerken GV, Seyfert HM (2002). A 3′-UTR variant of the inducible porcine hsp70.2 gene affects mRNA stability. Biochim Biophys Acta.

[30] Lee I, Ajay SS, Yook JI (2009). New class of microRNA targets containing simultaneous 5′-UTR and 3′-UTR interaction sites. Genome Res.

[31] Hou J, An X, Song Y, Gao TY, Lei YN, Cao BY (2015). Two mutations in the caprine MTHFR 3′UTR regulated by microRNAs are associated with milk production traits. PLoS One.

